# The role of primary care data in multimorbidity estimates using administrative health data

**DOI:** 10.1177/26335565261477731

**Published:** 2026-08-11

**Authors:** Clare Tazzeo, Katharina Schmidt-Mende, Karin Modig

**Affiliations:** 1 Division of Family Medicine and Primary Care, Department of Neurobiology, Care Sciences and Society, 27106 Karolinska Institutet, Huddinge, Sweden; 2 Unit of Epidemiology, Institute of Environmental Medicine, 27106 Karolinska Institutet, Stockholm, Sweden

**Keywords:** multimorbidity, primary care, hospital care, specialist outpatient care, data source

## Abstract

**Background:**

This study aims to investigate the extent to which two frequently used multimorbidity measures, the Charlson Comorbidity Index (CCI) and the 2+ count of chronic diseases, are underestimated when primary care data are absent, and to examine if this varies by sociodemographic factors.

**Methods:**

This register-based study included adults, aged 60 years and older, living in Stockholm, Sweden from 2017-2022 (n=497736). Multimorbidity was operationalized as 1) 2+ chronic diseases from a list of 60 and 2) CCI score of 2+. The multimorbidity prevalences were calculated using only outpatient specialist and hospital data, then after adding primary care data. The proportion of the prevalence underestimated without primary care data was estimated for both measures. Stratified analyses were conducted to assess the underestimation by age, sex, education, cohabitation, and care status.

**Findings:**

Without primary care data, approximately 20% of individuals with multimorbidity were missed (2+count: 19.4%,95%CI:19.3%-19.5%; 2+CCI: 23.9%,95%CI:23.8%-24.0%). The proportion of missed cases was highest among community-dwelling individuals (2+count: 21.0%,95%CI:20.8%-21.1%; 2+CCI: 25.5%,95%CI:25.3%-25.6%) and decreased with greater social care need (home care: 2+count: 4.33%,95%CI:4.09%-4.58%; 2+CCI: 16.0%,95%CI:15.5%-16.4%; nursing homes: 2+count: 7.78%,95%CI:7.35%-8.22%, 2+CCI: 18.0%,95%CI:17.3%-18.6%). The underestimation without primary care was greater among younger age groups for the 2+count, and women and older individuals for the 2+CCI.

**Interpretation:**

Multimorbidity is underestimated without primary care data, particularly among community-dwelling and independent older adults, as well as the youngest and oldest segments of the older population. Caution is warranted when comparing multimorbidity across age groups, care status, or social factors in the absence of primary care data.

## Introduction

Commonly defined as the co-occurrence of multiple chronic conditions in the same individual,^1^ multimorbidity is an important indicator of health. Its complex clinical management^2^ and burdensome outcomes like mortality,^3^ hospitalization,^4^ and disability^5^ make multimorbidity challenging for healthcare systems to manage. Population-level estimates of multimorbidity are essential for public health monitoring and resource allocation.^6^ Registers containing administrative health data are an attractive source for measuring health indicators like multimorbidity, as they are accessible, include far-reaching populations, and are routinely collected.^7^ However, many multimorbidity estimates are based only on hospital data.^8^ This is also the case for Sweden.^9,10^ Despite its many national registers on administrative health data, there is no nationwide primary care register. Fortunately, for several of the 21 different regions in Sweden responsible for administering primary care, including region Stockholm, administrative data on all primary care visits can be accessed through individual applications.^11^ With many chronic conditions being treated in primary care settings,^2,12^ it is certain that multimorbidity estimates based only on hospital and specialist data are underestimated; however, the extent of this is unknown. It is also not known if such underestimation is systematic or not.

Other studies^13–20^ have investigated how multimorbidity prevalence varies by data source, however, most have focused on differences by administrative, pharmaceutical, or self-reported data. Although some have included a primary care element,^13,14,20^ there is no study restricted to older adults that computes the underestimation in multimorbidity prevalence when specialist and hospital, but not primary, care data are used. Likewise, it remains unclear whether social factors lead to systematic differences in this underestimation. With the frequent inclusion of multimorbidity as a core measure or covariate in epidemiological research, it is essential to understand if and to what extent omitting primary care data biases estimates, particularly those involving sociodemographic elements. With population aging, accurate multimorbidity surveillance is critical for informing resource distribution and public health planning. Accordingly, this study aims to investigate the extent to which two of the most frequently used multimorbidity measures, namely the Charlson Comorbidity Index (CCI) and the 2+ count of chronic diseases, are underestimated when primary care data is absent, and to examine if this varies by sociodemographic factors.

## Methods

### Study design and participants

This register-based study included all residents of Stockholm, Sweden, who were alive and aged 60 years or older as of December 31^st^, 2022. In order to assess multimorbidity over the same time period for all participants, only those who had lived in Stockholm since January 2017 or earlier were included. Using the Total Population Register (TPR) of Sweden, 497 736 individuals matching these criteria were identified and included in the study. The Regional Ethics Committee in Stockholm approved this study (2011-136-31/5, 2022-03486-02) and informed consent was not required due to the register-based nature of this study.

### Procedures

Using the participants’ unique identification numbers, data from the TPR were linked to other Swedish registers to operationalize the multimorbidity and sociodemographic measures.

### Multimorbidity measures

Two of the most frequently used multimorbidity measures, across different levels of care, in the Swedish context and globally,^8,21,22^ and both with distinct diseases, cutoffs, weighting, and numbers of conditions, were computed. The first measure was defined as exhibiting two or more diseases from a pre-defined list of 60 categories of chronic diseases,^22^ and is hereby referred to as the ‘2+ count’ measure. These 60 disease categories were established by a group of experts who collected all international classification of diseases, 10th revision (ICD-10) codes corresponding to diseases that were chronic in nature, and then grouped them into categories based on clinical characteristics.^22^ The Charlson Comorbidity Index (CCI), a weighted multimorbidity measure involving 18 chronic disease categories with a score from 0 to 30, was the second measure included in this study, specifically a version tailored for use in the Swedish context.^23^ In the underestimation calculations, a CCI score of 2 or more, hereby referred to as ‘2+ CCI’, was used. In the percentage agreement calculations, the CCI score was not categorized, and thus, could take any whole number from 0 to 30.

To assess the degree to which multimorbidity prevalence is underestimated in the absence of primary care data, we computed both multimorbidity measures using 1) only primary care data, 2) only specialist outpatient care and hospital data, and 3) both primary care as well as specialist outpatient care and hospital data. Primary care data were ascertained through the Stockholm regional healthcare database (VAL).^11^ Specialist outpatient and hospital data were collected through the Swedish National Patient Register (NPR).^24^ ICD-10 codes, both main and secondary diagnoses, corresponding to the multimorbidity measures were obtained from these different sources between January 1st, 2017 and December 31st, 2022. Please refer to Appendices 1-2 for a list of the diseases and ICD-10 codes used in the 2+ count and CCI multimorbidity measures, respectively.

### Sociodemographic measures

Age on December 31^st^, 2022 was calculated from the TPR and categorized into 60-64, 65-69, 70-74, 75-79, 80-84, 85-89, and 90+ years. Sex (male or female) was also ascertained from the TPR. The Longitudinal Integration Database for Health Insurance and Labour Market Studies^25^ was used to identify educational attainment as of December 2022, which was categorized into primary (≤9 years), secondary (10-13 years), and tertiary (>13 years). Cohabitation status as of December 2022 was collected from the Dwelling Register and defined as living alone or cohabiting.^26^ Care status during the year 2022 was ascertained from the Social Service Register,^27^ and classified as living in a care home, receiving formal personal care at home (i.e., support in cleaning, dressing, eating), and community-dwelling without any formal care. Register data on ethnicity is not collected in Sweden; thus, it could not be included as a covariate.

### Statistical analyses

The prevalences (point prevalence on December 31^st^, 2022) of both multimorbidity measures were calculated using 1) only primary care data (VAL), 2) only outpatient specialist and hospital care data (NPR), and 3) a combination of both sources, using all available NPR and VAL data (NPR & VAL combined). In line with a method previously utilized by our group,^12^ the following was calculated to discern the proportion of the prevalence of these measures that is underestimated when primary care data is not used,

$$Underestimation\;of\;multimorbidity\;prevalence\;without\;primary\;care=1-\frac{{prevalence}_{Multimorbidity\;in\;NPR}}{{prevalence}_{Multimorbidity\;in\;NPR\;\&\;VAL\;combined}}$$

The above underestimation of multimorbidity is based on population-level estimates of prevalence. We also wanted to examine the concordance of multimorbidity measured with and without primary care on an individual level. To do this, we computed the percentage agreement, which represents the percentage of participants whose multimorbidity measure is the same when assessed using only hospital/specialist data (NPR) versus when using a combination of both sources. The percentage agreement was calculated as,

$$Percentage\;agreement=\frac{n\left({Multimorbid}_{NPR}={Multimorbid}_{NPR\;\&\;VAL\;combined}\right)}{n}$$

We also presented 2x2 tables to demonstrate the concordance and discordance in multimorbidity prevalence between primary care data and outpatient and specialist care data for both multimorbidity measures.

To assess whether systematic differences in the underestimation of multimorbidity without primary care are present across different subgroups, stratified analyses were conducted. The underestimation and percentage agreement calculations were both repeated, stratified by age group, sex, education, cohabitation, and care status. We calculated 95% confidence intervals (CIs) for all underestimation and percentage agreement calculations. Data linkage was performed in SAS version 9.4, and the analyses were conducted in Stata/SE 17.0 and R version 4.5.2.

### Sensitivity analyses

Two sensitivity analyses were conducted to assess the robustness of the findings. First, all analyses involving the 2+ count variable were repeated, using a cutoff of 3+ diseases and then a cutoff of 4+ diseases. Next, the analyses were repeated, restricting the population to those not receiving long-term care (personal home care or nursing home care).

### Role of the funding source

The funders of the study had no role in study design, data collection, data analysis, data interpretation, or writing of the report.

## Results

The median age of the 497 736 participants was 72 (IQR=13) years as of December 31st, 2022, and 53.6% of them were women (Table 1). Moreover, 82.2% of the participants received a secondary education or greater and 37.8% lived alone, as of December 31st, 2022. In addition, 40.9% of the participants received home care, while 22.7% lived in care homes during the year 2022.

**Table 1.** Characteristics of the study population as of December 31^st^, 2022 (n=497 736).

​	n	%
Age		
60-64	110 270	22.2
65-69	101 795	20.5
70-74	92 807	18.7
75-79	91 096	18.3
80-84	54 724	11.0
85-89	28 923	5.8
90-94	13 483	2.7
95+	4 638	0.9
Sex (female)	267 010	53.6
Education		
Primary	87 647	17.8
Secondary	198 036	40.3
Tertiary	205 781	41.9
Living alone	183 642	37.8
Care status^a^		
No formal care	23 587	36.4
Home care^b^	26 472	40.9
Care home	14 695	22.7

*Note.* Missing data: education (n=6272), cohabiting (n=638).

^a^Care status includes any occurrence during 2022.

^b^Home care refers to personal care services (i.e., support in cleaning, dressing, eating) at home.

**Figure 1.**
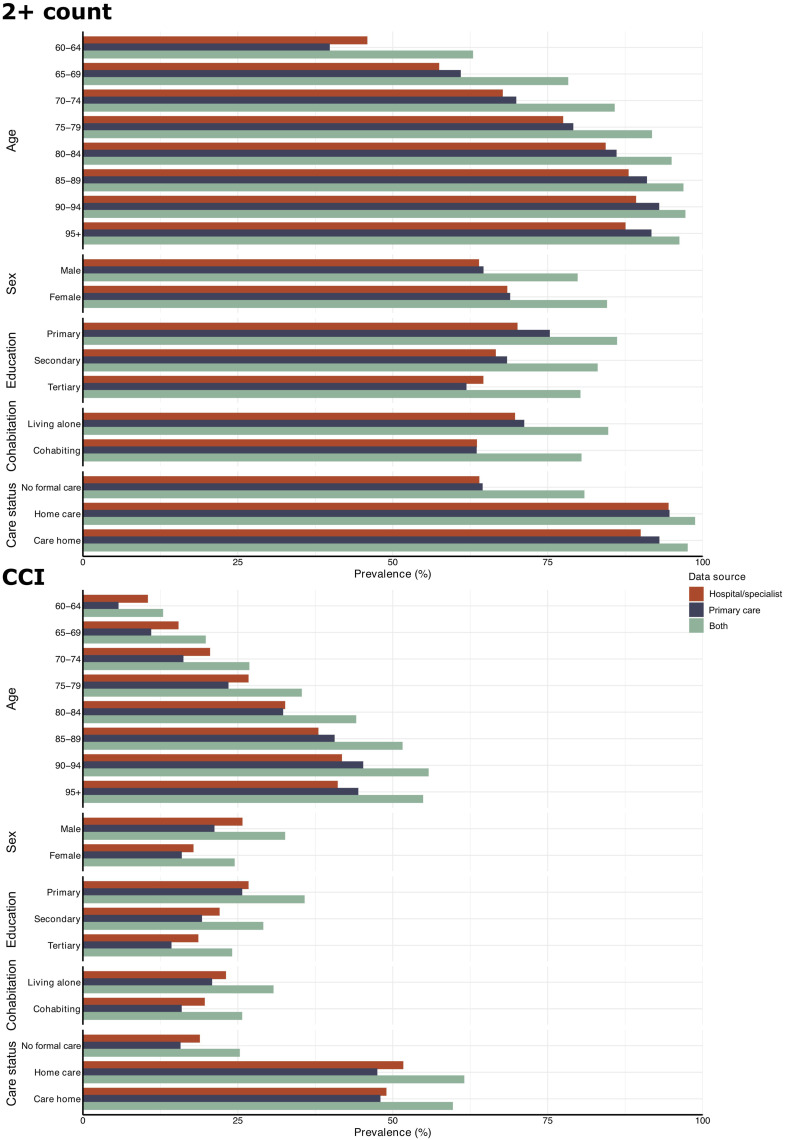
Prevalence of multimorbidity measured using hospital/specialist data, primary care data, and both sources across different sociodemographic characteristics. *Note.* CCI = Charlson comorbidity index.

The prevalence of the 2+ count measure was similar when measured using hospital/specialist data (66.4%) and when using primary care data (66.9%), with a substantially higher prevalence (82.4%) when both sources were utilized. Likewise, the prevalence of the 2+ CCI score was similar with hospital/specialist data (21.5%) and primary care data (18.4%), but higher (28.3%) when both sources were used. Table S1 presents the concordance and discordance of the multimorbidity measures when primary care versus outpatient specialist care/hospital data are used. The prevalence of multimorbidity by data source and sociodemographic factors are reported in Figure 1 and Table S2.

Without primary care data, both measures underestimated the prevalence of multimorbidity by around 20% (2+ count: 19.4%, 95% CI: 19.3%-19.5%, 2+ CCI: 23.9%, 95%CI: 23.8%-24.0%). In terms of concordance at the individual level, the percentage agreement analyses demonstrated that the multimorbidity scores of most of the participants (2+ count: 84.0%, 95%CI: 83.9%-84.1%; CCI: 82.0%, 95%CI: 81.9%-82.1%) were the same when both data sources were included versus when only inpatient/specialist data were utilized.

**Figure 2.**
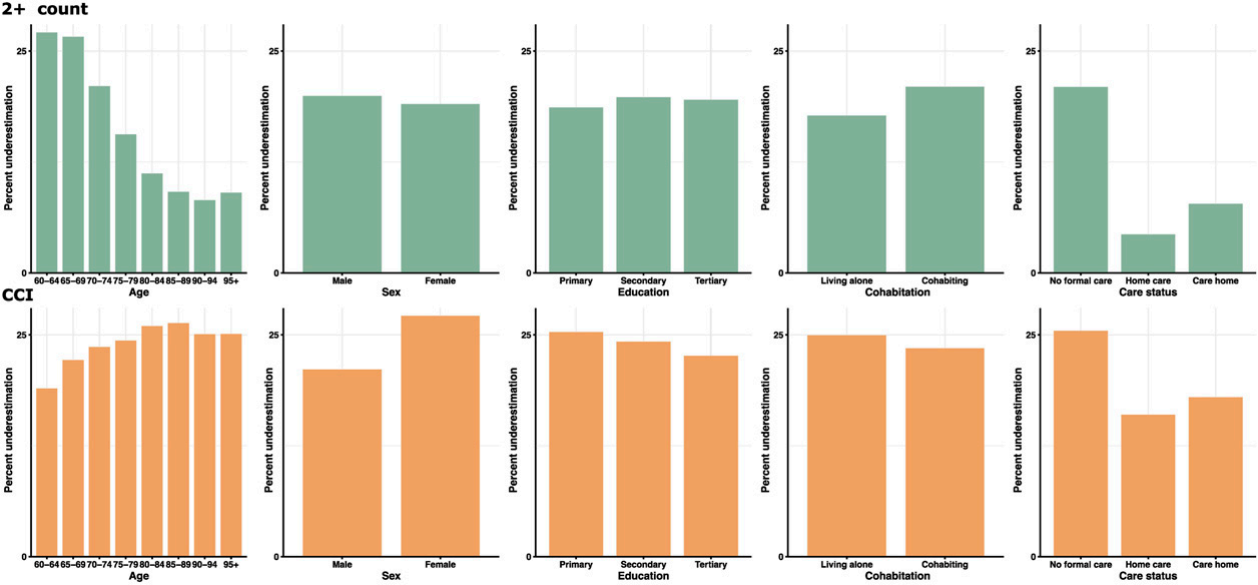
Percent of multimorbidity prevalence underestimated without primary care data stratified by sociodemographic characteristics. *Note.* CCI = Charlson comorbidity index.

The results of the stratified underestimation analyses are presented in Figure 2 and Table S3. For the 2+ count measure, the underestimation without primary care data was greater among younger age groups, ranging from an underestimation of 27.1% (95%CI: 26.8%-27.3%) to 8.19% (7.72%-8.65%) among those aged 60-64 and 90-94 years, respectively. In contrast, the 2+ CCI was underestimated more with increasing age, ranging from 18.9% (95%CI: 18.7%-19.2%) among those aged 60-64 years to 26.3% (95%CI: 25.8%-26.8%) among those aged 85-89 years, where it then levelled off. Regarding sex, excluding primary care data made little difference for the 2+ count. For the CCI 2+ measure, however, more women were missed than men, with underestimation rates of 27.1% (95%CI: 27.0%-27.3%) and 21.1% (95%CI: 20.9%-21.3%), respectively. When primary care data were excluded, the underestimation was similar across education levels for the 2+ disease count. Using the CCI 2+ measure, however, the underestimation was slightly higher among individuals with only primary education (25.3%, 95%CI: 25.0%-25.6%) compared to those with secondary (24.2%, 95%CI: 24.0%-24.4%) or tertiary (22.6%, 95%CI: 22.5%-22.8%) education. There was little difference by cohabitation status in the underestimation of multimorbidity measured by the 2+ CCI, while those cohabiting had a greater underestimation (21.0%, 95%CI: 20.8%-21.1%) compared to those living alone (17.7%, 95%CI: 17.6%-17.9%) for the 2+ count measure. For both multimorbidity definitions, when primary care data were missing, the underestimation was greatest among those without any formal long-term care (2+ count: 21.0%, 95%CI: 20.8%-21.1%; 2+ CCI: 25.5%, 95%CI: 25.3%-25.6%), followed by those in care homes (2+ count: 7.8%, 95%CI: 7.35%-8.22%; 2+ CCI: 18.0%, 17.3%-18.6%), and least in those receiving home care (2+ count: 4.33%, 95%CI: 4.09%-4.58%; 2+ CCI: 16.0%, 95%CI: 15.5%-16.4%).

**Figure 3.**
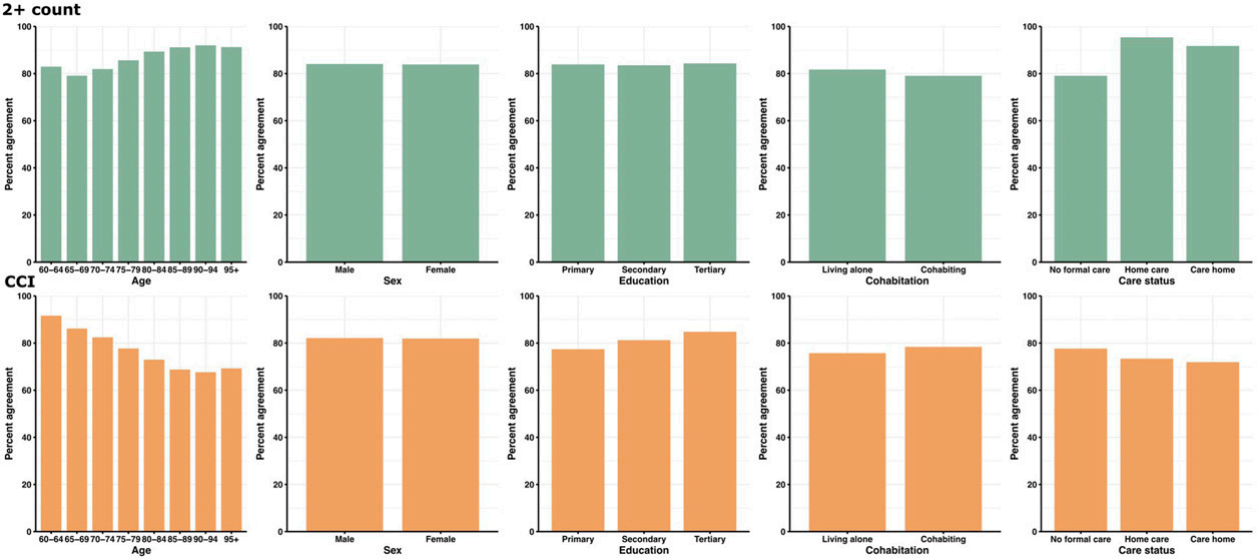
Percent agreement of multimorbidity presence using hospital/specialist data and both data sources (primary care and hospital/specialist data) stratified by sociodemographic characteristics. *Note.* CCI = Charlson comorbidity index.

Generally, the results of the percentage agreement analyses align with those of the above-described prevalence underestimations, across subgroups (Figure 3, Table S4). Those subgroups who experienced greater underestimations in their multimorbidity prevalence without primary care data also had a lower percentage of individuals that received the same multimorbidity score when both sources of data were used versus when only hospital/specialist data were included. There was a higher agreement in the presence of 2+ count multimorbidity between the hospital/specialist data and both sources at older ages (i.e., age 90-94: 92.0%, 95%CI: 91.6%-92.5%; age 60-64: 82.9%, 95%CI: 82.7%-83.2%) and among those receiving home care (95.4%, 95%CI: 95.1%-95.6%) and living in a care home (91.7%, 95%CI: 91.3%-92.2%), with little variation by sex, education, or living arrangement. In contrast, for the CCI, the agreement between hospital/specialist data versus both sources was higher among lower age groups (i.e., age 60-64: 91.6%, 95%CI: 91.5%-91.8%; age 90-94: 67.6%, 95%CI: 66.8%-68.4%), those with a higher education (i.e., tertiary: 84.8%, 95%CI: 84.6%-84.9%; primary: 77.4%, 95%CI: 77.1%-77.7%), and those not receiving any formal care (77.6%, 95%CI: 77.5%-77.8%), with less variation by sex and living arrangement.

The sensitivity analysis where the cut-off for the 2+ count measure was extended from 2+ to 3+ and 4+ revealed differences from the main analysis. The underestimation of multimorbidity prevalence without primary care data increased to 28.2% (95%CI: 28.0%-28.3%) with the 3+ diseases cutoff and 35.9% (95%CI: 35.8%-36.1%) with the 4+ diseases cutoff from 19.4% (95%CI: 19.3%-19.5%) with the 2+ cutoff. This trend of greater underestimation for the 3+ and 4+ diseases cutoffs held across the five sociodemographic characteristics (Table S5). Correspondingly, there was a decrease in the percentage of individuals with the same multimorbidity score when measured using both data sources versus only hospital/specialist data when the cutoff increased from 2+ (84.0%, 95%CI: 83.9%-84.1%) to 3+ (79.7%, 95%CI: 79.6-79.9) to 4+ (78.2%, 95%CI: 78.0%-78.3%) diseases. This was also reflected in the stratified analyses (Table S6). The sensitivity analysis restricted to those without long-term care revealed greater underestimations of the 2+ count (21.0%, 95%CI: 20.8%-21.1%) and 2+ CCI (25.5%, 95%CI: 25.3%-25.6%) compared to those of the total population. Correspondingly, the percentage of individuals with the same multimorbidity score when using both data sources versus hospital/specialist data was lower for both the 2+ count (79.1%, 95%CI: 78.9%-79.2%) and CCI (77.6%, 95%CI: 77.5%-77.8%) measures in this population not receiving long-term care. This trend of greater underestimation and lower percentage agreement without primary care data held across the different sociodemographic variables. (Table S7-8).

## Discussion

Leveraging data from the entire population of region Stockholm aged 60 years and older, our study reveals that omitting primary care data when measuring multimorbidity results in moderate underestimation. Both multimorbidity prevalences measured as a count and through the CCI were underestimated in the order of 19 to 24%. Despite clear concerns regarding the underestimation in the total population of older adults, the stratified analyses revealed that systematic differences by sociodemographic factors were less of an issue. However, more pronounced underestimations among the oldest and youngest segments of older adults, as well as those not receiving long-term care, highlight the importance of including primary care data for these groups.

The 2+ count and CCI, two of the most commonly used multimorbidity measures in the Swedish context and globally,^8,21,22^ were underestimated in the absence of primary care data. This aligns with other research^13,14,20^ that found multimorbidity prevalence estimates based on primary care data to be higher than those based on inpatient or self-reported data. Financial incentives to report more comorbidities in primary care and tendencies for only reporting comorbidities related to the clinical specialty in outpatient specialist care or limiting reporting to severe and acute conditions in hospital care may also explain these differences. In two studies,^13,14^ the difference in prevalence between both data sources and only hospital data was higher than in our study, however, unlike ours, these studies did not include specialist outpatient care in their hospital data source. The underestimation in the count measure may stem from primary care being the first point of contact for chronic infectious (i.e., Lyme disease), psychiatric, sleep, allergic, and cardiovascular conditions, as our previous study showed when primary care data were missing.^12^ Although burdensome on an individual and societal level, these diseases may be less likely to manifest as acute events and more likely to fall within the realm of primary care jurisdiction, hence why they are greatly underestimated without primary care data.^12,13^ The underestimation of multimorbidity driven by such diseases is problematic, as these diseases, especially in combination, are both burdensome and prevalent.^12^ Notably, this underestimation increases when the 2+ cutoff is extended to 3+ and 4+. This is important to point out, as many researchers utilize higher cutoff points in older populations, due to the larger proportion of individuals with at least two chronic diseases.^8^ Extra caution should be paid when using higher cut-off points without primary care data.

Interestingly, the underestimation of multimorbidity prevalence without primary care data was greater for the CCI than the 2+ count. Given that the CCI was underestimated most among the oldest adults (age 80+), this finding may be explained by the fact that diseases like dementia, liver disease, and kidney disease, all contributing strongly to the CCI, are often managed in primary care in the oldest ages.^12,28^ This finding indicates that the validity of the CCI as a multimorbidity measure among older adults becomes questionable without primary care data, particularly so in those aged 80+. This must be considered when using the CCI to describe the burden of multimorbidity among older adults or when comparing health status by age, as those who are 80+ will appear to be healthier than in reality. The opposite was found for the 2+ count measure, where the underestimation was greater at relatively younger ages. Another study reported higher multimorbidity prevalences among younger adults when primary care data is used instead of inpatient data.^13^ Hypertension, thyroid disease, diabetes, and osteoporosis are some of the conditions in the count variable that might drive this underestimation at younger ages, as they are often treated in primary care and consequently underestimated in relatively younger older adults.^12^ Regardless of the measure used, one must be careful in making any age-related forecasts or conclusions when omitting primary care data.

There was a considerably greater underestimation of both multimorbidity measures in the absence of primary care data among those who were community-dwelling and independent compared to those who were in care homes or receiving home care. This is also reflected in the sensitivity analysis excluding those receiving long-term care, which indicates that those who are not receiving long-term care really drive the underestimation of multimorbidity prevalence. This makes sense, as these individuals are less likely to be severely ill, and, thus, would receive most of their care in the primary care setting. While it is comforting that multimorbidity is not being underestimated in the frailest segment of the population, these results do highlight the importance of including primary care data when comparing multimorbidity by frailty or care status.

Fortunately, beyond the differences by age and care status, there was little variation in the underestimation of multimorbidity prevalence by other social factors. The only notable difference in the 2+ count was by living arrangement, with those who were cohabiting being more likely to be missed in the absence of primary care data. Perhaps those with a spouse or cohabitant are more encouraged to seek primary care, particularly for conditions that are typically not severe enough to necessitate hospitalization or specialist care, such as depression or sleep disorders.^12^ With the CCI, women and those with less education were more likely to be missed without primary care data. Another study also noted higher rates of multimorbidity among women when primary care data were included versus when only hospital data were used.^13^ This may be due to different care seeking patterns and diseases by education and sex,^29,30^ which lead to more management in the primary care setting for diseases in the CCI among these groups. Regardless of the reason for such differences, these results highlight the importance of being mindful of data source when drawing comparisons in multimorbidity status by these social factors in research, and particularly, when distributing health-related resources.

The comprehensive data on primary, specialist, and hospital care use across the entire population of older adults in Stockholm, as well as the two complementary methods to assess how multimorbidity estimates are influenced by the absence of primary care data, are strengths of this study. However, there are some limitations to note. First, there is a potential for misclassification of the chronic diseases, and thus, multimorbidity measures. This can occur through incorrect or missed coding of diagnoses in either register. Notably, a recent study demonstrated the validity of the NPR,^31^ increasing our confidence in the hospital/specialist care estimates. Moreover, we included diagnoses between January 1^st^, 2017 and December 31^st^ 2022 in our prevalence estimates; diagnoses identified during this six year period were assumed chronic and present on December 31^st^, 2022. It is possible, albeit unlikely, that some diagnoses were no longer present on December 31^st^, 2022. Likewise, we may have missed some diagnoses occurring before January 1^st^, 2017 that were still present at the time of the prevalence calculation. Next, there is a potential for selection bias due to missing data in the analyses stratified by education and cohabitation. However, the percentage of missing data in these measures is low (<2%) and likely does not pose a large issue. To continue, this study was partially conducted during the COVID-19 pandemic. Healthcare seeking patterns were different than normal during the pandemic,^32^ perhaps leading to underdiagnoses of chronic conditions, particularly those that were less severe. It is unclear how this may have influenced our estimates, however, the majority of the study period occurs before and beyond the first year of the COVID-19 pandemic, when healthcare seeking was most altered.^32^ Lastly, there are many other multimorbidity definitions besides those used in this study, each including different diseases; caution must be made when generalizing the results of this study to different multimorbidity measures and settings outside of Region Stockholm.

In this population-based study of all adults aged 60 years and older in Region Stockholm, we demonstrate that omitting primary care data leads to a consistent and, in certain subpopulations, more substantial underestimation of multimorbidity prevalence. Multimorbidity estimates based on both simple disease counts and the CCI were underestimated in the absence of primary care data, with the magnitude of underestimation increasing when higher disease count cut-offs were applied. The underestimation was particularly pronounced among community-dwelling and independent older adults, as well as among the youngest and oldest segments of the older population, depending on the multimorbidity measure used. As a result, studies relying solely on inpatient or specialist data risk mischaracterizing both the overall burden of multimorbidity and its distribution across age and care groups. Reassuringly, there were relatively few systematic differences in underestimation by most sociodemographic characteristics. Nevertheless, caution is warranted when comparing multimorbidity across age groups, care status, or social factors in the absence of primary care data.

## Supplemental material

Supplemental material - The role of primary care data in multimorbidity estimates using administrative health data Supplemental material for The role of primary care data in multimorbidity estimates using administrative health data by Clare Tazzeo, Katharina Schmidt-Mende, Karin Modig in Journal of Multimorbidity and Comorbidity

## Data Availability

The individual level data included in this study cannot be shared publicly due to the General Data Protection Regulation in Sweden. Access to the data and statistical code can be permitted to external researchers following ethical vetting and the establishment of a collaboration agreement. Contact the corresponding author for questions about data sharing.*
