# Supplementary material for: The role of primary care data in multimorbidity estimates using administrative health data
Source: J Multimorb Comorb. 2026 Aug 11;16:26335565261477731. doi: 10.1177/26335565261477731 (PMC13462637; doi:10.1177/26335565261477731)
Supplement: Supplementary file 1 [file sj-pdf-1-cob-10.1177_26335565261477731.pdf]

Supplementary Material

Page 2 – Table S1: Concordance and discordance between multimorbidity status across primary care and outpatient specialist care/hospital data for the MM 2+ and CCI 2+ measures

Page 3 – Table S1: Prevalence (%) of multimorbidity measured with hospital/specialist data, primary care data, and both sources across different sociodemographic characteristics

Page 4 – Table S2: Percent (%) of multimorbidity prevalence underestimated without primary care data stratified by sociodemographic characteristics

Page 5 – Table S3: Percent agreement (%) of multimorbidity presence using only hospital/specialist data and both hospital/specialist and primary care data stratified by sociodemographic characteristics

Page 6 – Table S4: Percent (%) of multimorbidity prevalence underestimated without primary care data stratified by sociodemographic characteristics and across different multimorbidity cutoffs

Page 7 – Table S5: Percent agreement (%) of multimorbidity presence using only hospital/specialist data and both hospital/specialist and primary care data stratified by sociodemographic characteristics and across different multimorbidity cutoffs

Page 8 – Table S6: Percent (%) of multimorbidity prevalence underestimated without primary care data stratified by sociodemographic characteristics in those without care

Page 9 – Table S7: Percent agreement (%) of multimorbidity presence using only hospital/specialist data and both hospital/specialist and primary care data stratified by sociodemographic characteristics in those without care

Pages 10-14 – Appendix 1: 60 chronic disease categories and corresponding ICD-10 codes in 2+ count multimorbidity measure

Page 15 – Appendix 2: Chronic diseases and ICD-10 codes included in Charlson Comorbidity Index

Table S1: Concordance and discordance between multimorbidity status across primary care and outpatient specialist care/hospital data for the MM 2+ and CCI 2+ measures

**MM 2+**			
		Primary care	
Outpatient specialist care/hospital		Multimorbid	Non-multimorbid
	Multimorbid	229 432	33 272
	Non-multimorbid	36 946	17 046
**CCI 2+**			
		Primary care	
Outpatient specialist care/hospital		Multimorbid	Non-multimorbid
	Multimorbid	63 247	65 279
	Non-multimorbid	45 844	282 199

Note. CCI = Charlson Comorbidity Index.

Table S2: Prevalence (%) of multimorbidity measured with hospital/specialist data, primary care data, and both sources across different sociodemographic characteristics

	2+ count			CCI 2+		
	**NPR**	**VAL**	**Both**	**NPR**	**VAL**	**Both**
**Age**						
**60-64**	45.9	39.8	63.0	10.5	5.75	12.9
**65-69**	57.5	61.0	78.3	15.4	11.0	19.8
**70-74**	67.8	69.9	85.8	20.5	16.2	26.9
**75-79**	77.5	79.1	91.8	26.7	23.5	35.3
**80-84**	84.4	86.1	95.0	32.6	32.3	44.1
**85-89**	88.1	91.0	96.9	38.0	40.6	51.6
**90-94**	89.3	93.0	97.2	41.8	45.2	55.8
**95+**	87.6	91.7	96.3	41.1	44.4	54.9
**Sex**						
**Male**	63.9	64.6	79.8	25.8	21.2	32.6
**Female**	68.5	68.9	84.6	17.9	16.0	24.5
**Education**						
**Primary**	70.1	75.3	86.2	26.7	25.7	35.8
**Secondary**	66.6	68.4	83.1	22.1	19.2	29.1
**Tertiary**	64.6	61.9	80.3	18.6	14.3	24.1
**Living arrangement**						
**Living alone**	69.7	71.2	84.8	23.1	20.9	30.8
**Cohabiting**	63.6	63.5	80.5	19.7	15.9	25.7
**Care status**[a]						
**No formal care**	64.0	64.5	80.9	18.9	15.8	25.3
**Home care**[b]	94.5	94.7	98.8	51.7	47.5	61.5
**Care home**	90.0	93.0	97.6	49.0	48.0	59.7

Note. NPR = National Patient Register, VAL = Stockholm regional healthcare database, CCI = Charlson Comorbidity Index. Most recent measurement of each sociodemographic characteristic as of December 2022 is used. [a]Care status includes any occurrence during 2022. [b]Home care refers to personal care services (i.e., support in cleaning, dressing, eating) at home.

Table S3: Percent (%) of multimorbidity prevalence underestimated without primary care data stratified by sociodemographic characteristics

	2+ count	CCI 2+
	**% underestimated (95%CI)**	**% underestimated (95%CI)**
**Age**		
**60-64**	27.1 (26.8-27.3)	18.9 (18.7-19.2)
**65-69**	26.6 (26.3-26.9)	22.1 (21.9-22.4)
**70-74**	21.1 (20.8-21.3)	23.6 (23.3-23.9)
**75-79**	15.6 (15.4-15.9)	24.3 (24.1-24.6)
**80-84**	11.2 (10.9-11.5)	26.0 (25.6-26.4)
**85-89**	9.13 (8.80-9.46)	26.3 (25.8-26.8)
**90-94**	8.19 (7.72-8.65)	25.1 (24.3-25.8)
**95+**	9.03 (8.20-9.85)	25.1 (23.9-26.3)
**Sex**		
**Male**	19.9 (19.8-20.1)	21.1 (20.9-21.3)
**Female**	19.0 (18.9-19.2)	27.1 (27.0-27.3)
**Education**		
**Primary**	18.6 (18.4-18.9)	25.3 (25.0-25.6)
**Secondary**	19.8 (19.6-20.0)	24.2 (24.0-24.4)
**Tertiary**	19.5 (19.3-19.7)	22.6 (22.5-22.8)
**Living arrangement**		
**Living alone**	17.7 (17.6-17.9)	24.9 (24.7-25.1)
**Cohabiting**	21.0 (20.8-21.1)	23.5 (23.3-23.6)
**Care status[a]**		
**No formal care**	21.0 (20.8-21.1)	25.5 (25.3-25.6)
**Home care[b]**	4.33 (4.09-4.58)	16.0 (15.5-16.4)
**Care home**	7.78 (7.35-8.22)	18.0 (17.3-18.6)

Note. VAL = Stockholm regional healthcare database, CCI = Charlson Comorbidity Index, 95%CI = 95% confidence interval. Most recent measurement of each sociodemographic characteristic as of December 2022 is used. [a]Care status includes any occurrence during 2022. [b]Home care refers to personal care services (i.e., support in cleaning, dressing, eating) at home.

Table S4: Percent agreement (%) of multimorbidity presence using only hospital/specialist data and both hospital/specialist and primary care data stratified by sociodemographic characteristics

	2+ count	CCI
	**Percent agreement (95% CI)**	**Percent agreement (95% CI)**
**Age**		
**60-64**	82.9 (82.7-83.2)	91.6 (91.5-91.8)
**65-69**	79.1 (78.9-79.4)	86.2 (85.7-86.4)
**70-74**	81.9 (81.7-82.2)	82.4 (82.2-82.7)
**75-79**	85.6 (85.4-85.9)	77.7 (77.4-78.0)
**80-84**	89.4 (89.1-89.6)	73.0 (72.6-73.3)
**85-89**	91.1 (90.8-91.5)	68.8 (68.2-69.3)
**90-94**	92.0 (91.6-92.5)	67.6 (66.8-68.4)
**95+**	91.3 (90.5-92.1)	69.3 (67.9-70.6)
**Sex**		
**Male**	84.1 (83.9-84.2)	82.1 (82.0-82.3)
**Female**	83.9 (83.8-84.0)	81.9 (81.8-82.1)
**Education**		
**Primary**	83.9 (83.6-84.2)	77.4 (77.1-77.7)
**Secondary**	83.6 (83.4-83.7)	81.2 (81.0-81.4)
**Tertiary**	84.3 (84.2-84.5)	84.8 (84.6-84.9)
**Living arrangement**		
**Living alone**	81.8 (81.6-82.0)	75.7 (75.5-75.9)
**Cohabiting**	79.0 (78.9-79.2)	78.4 (78.2-78.5)
**Care status**[a]		
**No formal care**	79.1 (78.9-79.2)	77.6 (77.5-77.8)
**Home care**[b]	95.4 (95.1-95.6)	73.4 (72.9-73.9)
**Care home**	91.7 (91.3-92.2)	71.9 (71.2-72.6)

Note. NPR = National Patient Register, VAL = Stockholm regional healthcare database, CCI = Charlson Comorbidity Index, 95%CI = 95% confidence interval. Most recent measurement of each sociodemographic characteristic as of December 2022 is used. [a]Care status includes any occurrence during 2022. [b]Home care refers to personal care services (i.e., support in cleaning, dressing, eating) at home.

Table S5: Percent (%) of multimorbidity prevalence underestimated without primary care data stratified by sociodemographic characteristics and across different multimorbidity cutoffs

	2+ count	3+ count	4+ count
**Age**			
**60-64**	27.1 (26.8-27.3)	36.8 (36.5-37.1)	44.1 (43.8-44.4)
**65-69**	26.6 (26.3-26.9)	37.2 (36.9-37.5)	45.7 (45.4-46.0)
**70-74**	21.1 (20.8-21.3)	31.3 (31.0-31.6)	40.5 (40.2-40.8)
**75-79**	15.6 (15.4-15.9)	24.9 (24.6-25.2)	33.8 (33.5-34.1)
**80-84**	11.2 (10.9-11.5)	19.2 (18.9-19.5)	27.6 (27.2-27.9)
**85-89**	9.13 (8.80-9.46)	16.3 (15.9-16.8)	24.1 (23.6-24.6)
**90-94**	8.19 (7.72-8.65)	14.3 (13.7-14.9)	20.9 (20.2-21.5)
**95+**	9.03 (8.20-9.85)	14.3 (13.3-15.3)	20.8 (19.6-21.9)
**Sex**			
**Male**	19.9 (19.8-20.1)	28.0 (27.9-28.2)	34.8 (34.6-35.0)
**Female**	19.0 (18.9-19.2)	28.3 (28.1-28.5)	36.8 (36.7-37.0)
**Education**			
**Primary**	18.6 (18.4-18.9)	26.4 (26.1-26.7)	33.6 (33.3-33.9)
**Secondary**	19.8 (19.6-20.0)	28.6 (28.4-28.8)	36.2 (36.0-36.4)
**Tertiary**	19.5 (19.3-19.7)	28.8 (28.6-29.0)	37.0 (36.8-37.2)
**Living arrangement**			
**Living alone**	17.7 (17.6-17.9)	26.1 (25.9-26.3)	33.8 (33.6-34.0)
**Cohabiting**	21.0 (20.8-21.1)	30.2 (30.1-30.4)	38.3 (38.2-38.5)
**Care status[a]**			
**No formal care**	21.0 (20.8-21.1)	30.5 (30.3-30.6)	39.0 (38.8-39.1)
**Home care[b]**	4.33 (4.09-4.58)	8.16 (7.83-8.49)	13.1 (12.7-13.5)
**Care home**	7.78 (7.35-8.22)	12.6 (12.0-13.1)	17.4 (16.8-18.0)

Note. VAL = Stockholm regional healthcare database, CCI = Charlson Comorbidity Index, 95%CI = 95% confidence interval. Most recent measurement of each sociodemographic characteristic as of December 2022 is used. [a]Care status includes any occurrence during 2022. [b]Home care refers to personal care services (i.e., support in cleaning, dressing, eating) at home.

Table S6: Percent agreement (%) of multimorbidity presence using only hospital/specialist data and both hospital/specialist and primary care data stratified by sociodemographic characteristics and across different multimorbidity cutoffs

	2+ count	3+ count	4+ count
	**Percent agreement (95% CI)**	**Percent agreement (95% CI)**	**Percent agreement (95% CI)**
**Age**			
**60-64**	82.9 (82.7-83.2)	82.8 (82.6-83.0)	85.3 (85.1-85.5)
**65-69**	79.1 (78.9-79.4)	75.9 (75.7-76.2)	76.7 (76.4-77.0)
**70-74**	81.9 (81.7-82.2)	76.5 (76.2-76.8)	74.5 (74.2-74.8)
**75-79**	85.6 (85.4-85.9)	79.0 (78.7-79.2)	74.8 (74.5-75.0)
**80-84**	89.4 (89.1-89.6)	82.7 (82.3-83.0)	77.1 (76.7-77.4)
**85-89**	91.1 (90.8-91.5)	84.7 (84.3-85.1)	78.7 (78.3-79.2)
**90-94**	92.0 (91.6-92.5)	86.4 (85.8-87.0)	81.2 (80.6-81.9)
**95+**	91.3 (90.5-92.1)	86.6 (85.7-87.6)	81.7 (80.5-82.8)
**Sex**			
**Male**	84.1 (83.9-84.2)	80.7 (80.5-80.9)	80.0 (79.8-80.2)
**Female**	83.9 (83.8-84.0)	78.9 (78.8-79.1)	76.6 (76.4-76.7)
**Education**			
**Primary**	83.9 (83.6-84.2)	79.5 (79.2-79.7)	77.1 (76.8-77.4)
**Secondary**	83.6 (83.4-83.7)	79.2 (79.1-79.4)	77.6 (77.5-77.8)
**Tertiary**	84.3 (84.2-84.5)	80.3 (80.1-80.4)	79.0 (78.9-79.2)
**Living arrangement**			
**Living alone**	81.8 (81.6-82.0)	80.3 (80.1-80.5)	77.9 (77.7-78.1)
**Cohabiting**	79.0 (78.9-79.2)	79.1 (79.0-79.3)	78.1 (78.0-78.3)
**Care status[a]**			
**No formal care**	79.1 (78.9-79.2)	78.8 (78.6-78.9)	77.4 (77.3-77.5)
**Home care[b]**	95.4 (95.1-95.6)	92.1 (91.7-92.4)	87.8 (87.4-88.1)
**Care home**	91.7 (91.3-92.2)	88.2 (87.6-88.7)	84.7 (84.1-85.3)

Note. NPR = National Patient Register, VAL = Stockholm regional healthcare database, CCI = Charlson Comorbidity Index, 95%CI = 95% confidence interval. Most recent measurement of each sociodemographic characteristic as of December 2022 is used. [a]Care status includes any occurrence during 2022. [b]Home care refers to personal care services (i.e., support in cleaning, dressing, eating) at home.

Table S7: Percent (%) of multimorbidity prevalence underestimated without primary care data stratified by sociodemographic characteristics in those without care

	2+ count		CCI 2+	
	**Total pop**	**No care**	**Total pop**	**No care**
**Age**				
**60-64**	27.1 (26.8-27.3)	27.4 (27.1-27.6)	18.9 (18.7-19.2)	19.2 (19.0-19.5)
**65-69**	26.6 (26.3-26.9)	27.1 (26.8-27.4)	22.1 (21.9-22.4)	22.7 (22.5-23.0)
**70-74**	21.1 (20.8-21.3)	21.8 (21.5-22.0)	23.6 (23.3-23.9)	24.6 (24.3-24.9)
**75-79**	15.6 (15.4-15.9)	16.5 (16.2-16.7)	24.3 (24.1-24.6)	26.0 (25.7-26.3)
**80-84**	11.2 (10.9-11.5)	12.3 (12.0-12.6)	26.0 (25.6-26.4)	28.7 (28.3-29.2)
**85-89**	9.13 (8.80-9.46)	11.0 (10.5-11.4)	26.3 (25.8-26.8)	31.2 (30.6-31.8)
**90-94**	8.19 (7.72-8.65)	11.5 (10.7-12.3)	25.1 (24.3-25.8)	32.6 (31.4-33.7)
**95+**	9.03 (8.20-9.85)	12.6 (10.8-14.4)	25.1 (23.9-26.3)	34.3 (31.8-36.9)
**Sex**				
**Male**	19.9 (19.8-20.1)	21.2 (21.0-21.3)	21.1 (20.9-21.3)	22.5 (22.3-22.6)
**Female**	19.0 (18.9-19.2)	20.8 (20.6-20.9)	27.1 (27.0-27.3)	29.4 (29.2-29.6)
**Education**				
**Primary**	18.6 (18.4-18.9)	20.9 (20.6-21.2)	25.3 (25.0-25.6)	28.0 (27.7-28.3)
**Secondary**	19.8 (19.6-20.0)	21.3 (21.1-21.5)	24.2 (24.0-24.4)	25.9 (25.7-26.1)
**Tertiary**	19.5 (19.3-19.7)	20.6 (20.5-20.8)	22.6 (22.5-22.8)	23.6 (23.4-23.7)
**Living arrangement**				
**Living alone**	17.7 (17.6-17.9)	19.8 (19.6-20.0)	24.9 (24.7-25.1)	27.3 (27.1-27.6)
**Cohabiting**	21.0 (20.8-21.1)	21.6 (21.5-21.8)	23.5 (23.3-23.6)	24.4 (24.2-24.5)

Note. VAL = Stockholm regional healthcare database, CCI = Charlson Comorbidity Index, 95%CI = 95% confidence interval. Most recent measurement of each sociodemographic characteristic as of December 2022 is used.

Table S8: Percent agreement (%) of multimorbidity presence using only hospital/specialist data and both hospital/specialist and primary care data stratified by sociodemographic characteristics in those without care

	2+ count		CCI	
	**Total pop**	**No care**	**Total pop**	**No care**
	**Percent agreement (95% CI)**	**Percent agreement (95% CI)**	**Percent agreement (95% CI)**	**Percent agreement (95% CI)**
**Age**				
**60-64**	82.9 (82.7-83.2)	76.6 (76.3-76.8)	91.6 (91.5-91.8)	83.7 (83.4-83.9)
**65-69**	79.1 (78.9-79.4)	73.6 (73.3-73.9)	86.2 (85.7-86.4)	80.1 (79.8-80.3)
**70-74**	81.9 (81.7-82.2)	77.8 (77.5-78.1)	82.4 (82.2-82.7)	77.7 (77.4-78.0)
**75-79**	85.6 (85.4-85.9)	82.8 (82.5-83.0)	77.7 (77.4-78.0)	74.6 (74.3-74.9)
**80-84**	89.4 (89.1-89.6)	86.9 (86.6-87.2)	73.0 (72.6-73.3)	70.7 (70.3-71.1)
**85-89**	91.1 (90.8-91.5)	88.5 (88.0-88.9)	68.8 (68.2-69.3)	66.4 (65.7-67.0)
**90-94**	92.0 (91.6-92.5)	88.1 (87.3-88.9)	67.6 (66.8-68.4)	63.7 (62.5-64.9)
**95+**	91.3 (90.5-92.1)	87.5 (85.7-89.3)	69.3 (67.9-70.6)	65.8 (63.2-68.4)
**Sex**				
**Male**	84.1 (83.9-84.2)	79.1 (79.0-79.3)	82.1 (82.0-82.3)	77.2 (77.0-77.4)
**Female**	83.9 (83.8-84.0)	79.0 (78.8-79.2)	81.9 (81.8-82.1)	78.0 (77.9-78.2)
**Education**				
**Primary**	83.9 (83.6-84.2)	78.8 (78.5-78.8)	77.4 (77.1-77.7)	72.4 (72.1-72.7)
**Secondary**	83.6 (83.4-83.7)	78.6 (78.5-78.8)	81.2 (81.0-81.4)	76.6 (76.4-76.8)
**Tertiary**	84.3 (84.2-84.5)	79.5 (79.3-79.6)	84.8 (84.6-84.9)	80.7 (80.5-80.9)
**Living arrangement**				
**Living alone**	81.8 (81.6-82.0)	80.0 (79.8-80.2)	75.7 (75.5-75.9)	76.1 (75.9-76.3)
**Cohabiting**	79.0 (78.9-79.2)	78.5 (78.4-78.7)	78.4 (78.2-78.5)	78.5 (78.3-78.6)

Note. NPR = National Patient Register, VAL = Stockholm regional healthcare database, CCI = Charlson Comorbidity Index, 95%CI = 95% confidence interval. Most recent measurement of each sociodemographic characteristic as of December 2022 is used.

Appendix 1: 60 chronic disease categories and corresponding ICD-10 codes in 2+ count multimorbidity measure

Chronic disease category	ICD-10 codes
Allergy	**Included:**<br>J301, J302, J303, J304, J450, K522, L20, L23, L500, Z516
Anemia	**Included:**<br>D50, D51, D52, D53, D55, D56, D57, D58, D59, D60, D61, D63, D64<br>**Excluded:**<br>D563, D590, D595, D593, D596, D601, D611, D612, D642
Asthma	**Included:**<br>J45
Atrial fibrillation	**Included:**<br>I48
Autoimmune diseases	**Included:**<br>I731, L10, L12, L40, L41, L93, L94, L95, M30, M31, M32, M33, M34, M35, M36<br>**Excluded:**<br>L105, M320, M342, M357, M358, M359, M360, M361, M362, M363
Blindness, visual impairment	**Included:**<br>H54, Z442, Z970<br>**Excluded:**<br>H543
Blood and blood forming organ diseases	**Included:**<br>D66, D67, D68, D69, D71, D720, D730, D731, D732, D74, D750, D761, D763, D77, D80, D81, D82, D83, D84, D86, D89<br>**Excluded:**<br>D683, D684, D695, D748, D807, D891, D893
Bradycardias and conduction diseases	**Included:**<br>I441, I442, I443, I453, I455, Z950
Cardiac valve diseases	**Included:**<br>I05, I06, I07, I08, I091, I098, I34, I35, I36, I37, I38, I390, I391, I392, I393, I394, Q22, Q23, Z952, Z953, Z954
Cataract and other lens diseases	**Included:**<br>H25, H26, H27, H28, Q12, Z961
Cerebrovascular disease	**Included:**<br>G45, G46, I60, I61, I62, I63, I64, I67, I69
Chromosomal abnormalities	**Included:**<br>Q90, Q91, Q92, Q93, Q95, Q96, Q97, Q98, Q99
Chronic infectious diseases	**Included:**<br>A15, A16, A17, A18, A19, A30, A31, A50, A52, A53, A65, A66, A67, A692, A81, B20, B21, B22, B23, B24, B381, B391, B401, B572, B573, B574, B575, B65, B92, B94, J65, M863, M864, M865, M866
Chronic kidney diseases	**Included:**<br>I120, I130, I131, I132, I139, N01, N03, N04, N05, N07, N08, N11, N183, N184, N185, N189, Q60, Q611, Q612, Q613, Q614, Q615, Q618, Q619, Z905, Z940
Chronic liver diseases	**Included:**<br>B18, K70, K713, K714, K715, K717, K721, K73, K74, K753, K754, K758, K761, K766, K767, K778, Q446, Z944<br>**Excluded:**<br>K700, K701
Chronic pancreas, biliary tract, and gallbladder diseases	**Included:**<br>K800, K801, K802, K808, K811, K86, Q440, Q441, Q442, Q443, Q444, Q445, Q450<br>**Excluded:**<br>K862, K863, K869
Chronic ulcer of the skin	**Included:**<br>I830, I832, L89, L97, L984

Colitis and related diseases	**Included:**<br>K520, K528, K551, K552, K572, K573, K574, K575, K578, K579, K58, K590, K592, K62, K634, K64<br><br>**Excluded:**<br>K620, K621, K625, K626, K645
COPD, emphysema, chronic bronchitis	**Included:**<br>J41, J42, J43, J44, J47
Deafness, hearing impairment	**Included:**<br>H80, H90, H911, H913, H919, Q16, Z453, Z461, Z962, Z974
Dementia	**Included:**<br>F00, F01, F02, F03, F051, G30, G31
Depression and mood diseases	**Included:**<br>F30, F31, F32, F33, F34, F38, F39, F412
Diabetes	**Included:**<br>E10, E11, E13, E14, E891
Dorsopathies	**Included:**<br>M40, M41, M42, M43, M47, M48, M49, M50, M51, M53, Q675, Q761, Q764
Dyslipidemia	**Included:**<br>E78
Ear, nose, throat diseases	**Included:**<br>H604, H661, H662, H663, H701, H71, H731, H741, H810, H831, H832, H95, J300, J31, J32, J33, J341, J342, J343, J35, J37, J380, J386, K051, K053, K07, K110, K117, Q30, Q31, Q32, Q35, Q36, Q37, Q38
Epilepsy	**Included:**<br>G40<br>**Excluded:**<br>G405
Esophagus, stomach and duodenum diseases	**Included:**<br>I85, I864, I982, I983, K21, K220, K222, K224, K225, K227, K230, K231, K254, K255, K256, K257, K264, K265, K266, K267, K274, K275, K276, K277, K284, K285, K286, K287, K293, K294, K295, K296, K297, K298, K299, K311, K312, K313, K314, K315, Q39, Q40, Z903
Glaucoma	**Included:**<br>H401, H402, H403, H404, H405, H406, H408, H409
Heart failure	**Included:**<br>I110, I130, I132, I27, I280, I42, I43, I50, I515, I517, I528, Z941, Z943
Hematological neoplasms	**Included:**<br>C81, C82, C83, C84, C85, C86, C88, C90, C91, C92, C93, C94, C95, C96
Hypertension	**Included:**<br>I10, I11, I12, I13, I15
Inflammatory arthropathies	**Included:**<br>M023, M05, M06, M07, M08, M09, M10, M11, M12, M13, M14, M45, M460, M461, M468, M469
Inflammatory bowel diseases	**Included:**<br>K50, K51
Ischemic heart disease	**Included:**<br>I20, I21, I22, I24, I25, Z951, Z955
Migraine and facial pain syndromes	**Included:**<br>G43, G440, G441, G442, G443, G448, G50
Multiple sclerosis	**Included:**<br>G35

Neurotic, stress-related and somatoform diseases	**Included:**<br>F40, F41, F42, F43, F44, F45, F48<br>**Excluded:**<br>F430, F432
Obesity	**Included:**<br>E66
Osteoarthritis and other degenerative joint diseases	**Included:**<br>M15, M16, M17, M18, M19, M362, M363
Osteoporosis	**Included:**<br>M80, M81, M82
Other cardiovascular diseases	**Included:**<br>I09, I281, I310, I311, I456 I495, I498, I70, I71, I72, I790, I791, I950, I951, I958, Q20, Q21, Q24, Q25, Q26, Q27, Q28, Z958, Z959<br><br>**Excluded:**<br>I091, I098, I702
Other digestive diseases	**Included:**<br>K660, K900, K901, K902, K911, K93, Q41, Q42, Q43, R15, Z904, Z980
Other eye diseases	**Included:**<br>H022, H023, H024, H025, H04, H05, H104, H17, H184, H185, H186, H187, H188, H189, H193, H198, H201, H21, H310, H311, H312, H318, H319, H33, H352, H353, H354, H355, H357, H358, H359, H36, H47, H48, H49, H51, Q10, Q11, Q13, Q14, Q15, Z947<br>**Excluded:**<br>H043, H050, H470, H471, H481
Other genitourinary diseases	**Included:**<br>B901, N200, N202, N209, N210, N218, N219, N22, N301, N302, N303, N304, N31, N320, N323, N328, N329, N33, N35, N393, N394, N480, N484, N489, N701, N711, N731, N734, N736, N761, N763, N81, N88, N895, N905, N952, Q54, Q620, Q621, Q622, Q623, Q624, Q627, Q628, Q638, Q639, Q640, Q641, Q643, Q644, Q645, Q646, Q647, Q648, Q649, Z906, Z907, Z960
Other metabolic diseases	**Included:**<br>E20, E21, E22, E23, E24, E25, E26, E27, E28, E29, E31, E34, E35, E40, E41, E42, E43, E44, E45, E46, E64, E70, E71, E72, E74, E75, E76, E77, E79, E80, E83, E84, E85, E88, E89, K903, K904, K908, K909, K912, M83, M88, N25<br>**Excluded:**<br>E231, E242, E244, E273, E343, E344, E350, E441, E790, E804, E883, E890, E892
Other musculoskeletal and joint diseases	**Included:**<br>B902, M212, M213, M214, M215, M216, M217, M218, M219, M22, M23, M24, M252, M253, M357, M61, M652, M653, M654, M700, M720, M722, M724, M750, M751, M753, M754, M797, M841, M89, M91, M93, M94, M96, M99, Q65, Q66, Q68, Q71, Q72, Q73, Q74, Q77, Q78, Q796, Q798, Q87, S382, S48, S58, S68, S78, S88, S98, T05, T096, T116, T136, T147, T90, T91, T92, T93, T94, T95, T96, T97, T98, Z440, Z441, Z891, Z892, Z893, Z894, Z895, Z896, Z897, Z898, Z899, Z946, Z966, Z971
Other neurological diseases	**Included:**<br>B900, D482, G041, G09, G10, G11, G12, G13, G24, G25, G26, G32, G37, G51, G52, G53, G70, G71, G723, G724, G728, G729, G73, G80, G81, G82, G83, G90, G91, G938, G939, G95, G99, M471, Q00, Q01, Q02, Q03, Q04, Q05, Q06, Q07, Q760<br>**Excluded:**<br>G130, G131, G251, G254, G256, G510, G732, G733, G734, G838
Other psychiatric and behavioural diseases	**Included:**<br>F04, F06, F07, F09, F102, F106, F107, F112, F116, F117, F122, F126, F127, F132, F136, F137, F142, F146, F147, F152, F156, F157, F162, F166, F167, F172, F176, F177, F182, F186, F187, F192, F196, F197, F50, F52, F60, F61, F62, F63, F68, F70, F71, F72, F73, F78, F79, F80, F81, F82, F83, F84, F88, F89, F95, F99

Other respiratory diseases	**Included:**<br>B909, E662, J60, J61, J62, J63, J64, J65, J66, J67, J684, J701, J703, J704, J84, J92, J941, J953, J955, J961, J98, Q33, Q34, Z902, Z942, Z943, Z963<br>**Excluded:**<br>J981
Other skin diseases	**Included:**<br>L13, L28, L301, L43, L508, L581, L85, Q80, Q81, Q821, Q822, Q829<br>**Excluded:**<br>L432
Parkinson and parkinsonism	**Included:**<br>G20, G21, G22, G23<br>**Excluded:**<br>G210
Peripheral neuropathy	**Included:**<br>B91, G14, G54, G55, G56, G57, G58, G59, G60, G628, G629, G63, M472, M531, M541<br>**Excluded:**<br>G631
Peripheral vascular disease	**Included:**<br>I702, I73, I792, I798<br>**Excluded:**<br>I731, I738
Prostate diseases	**Included:**<br>N40<br>N411<br>N418
Schizophrenia and delusional diseases	**Included:**<br>F20, F22, F24, F25, F28
Sleep disorders	**Included:**<br>F510, F511, F512, F513, G47
Solid neoplasms	**Included:**<br>C, D00, D01, D02, D03, D04, D05, D06, D07, D09, D320, D321, D329, D330, D331, D332, D333, D334, Q85<br>**Excluded:**<br>C81, C82, C83, C84, C85, C86, C88, C90, C91, C92, C93, C94, C95, C96
Thyroid diseases	**Included:**<br>E00, E01, E02, E03, E05, E062, E063, E065, E07, E350, E890<br>**Excluded:**<br>E035
Venous and lymphatic diseases	**Included:**<br>I780, I83, I87, I89, I972, Q820

Note. Main and secondary diagnoses are included. Adapted from Calderón-Larrañaga et al (1).

Appendix 2: Chronic diseases and ICD-10 codes included in Charlson Comorbidity Index

Chronic disease	ICD-10 codes	Weight in CCI score
Myocardial infarction	I21, I22, I252	1
Congestive heart failure	I110, I130, I132, I255, I420, I426, I427, I428, I429, I43, I50	1
Peripheral vascular disease	I70, I71, I731, I738, I739, I771, I790, I792, K55	1
Cerebrovascular disease	G45, I60, I61, I62, I63, I64, I67, I69	1
Chronic obstructive pulmonary disease	J43, J44	1
Other chronic pulmonary diseases	J41, J42, J45, J46, J47, J60, J61, J62, J63, J64, J65, J66, J67, J68, J69, J70	1
Rheumatic diseases	M05, M06, M123, M070, M071, M072, M073, M08, M13, M30, M313, M314, M315, M316, M32, M33, M34, M350, M351, M353, M45, M46	1
Dementia	F00, F01, F02, F03, F051, G30, G311, G319	1
Hemiplegia, tetraplegia	G114, G80, G81, G82, G830, G831, G832, G833, G838	2
Diabetes	E100, E101, E110, E111, E120, E121, E130, E131, E140, E141	1[a]
Diabetes with end-stage organ damage	E102, E103, E104, E105, E107, E112, E113, E114, E115, E116, E117, E122, E123, E124, E125, E126, E127, E132, E133, E134, E135, E136, E137, E142, E143, E144, E145, E146, E147	2
Moderate or severe kidney disease	I120, I131, N032, N033, N034, N035, N036, N037, N052, N053, N054, N055, N056, N057, N11, N18, N19, N250, Q611, Q612, Q613, Q614, Z49, Z940, Z992	2
Mild liver disease	B15, B16, B17, B18, B19, K703, K709, K73, K746, K754	1[b]
Moderate or severe liver disease	I850, I859, I982, I983 or R18 + (B15, B16, B17, B18, B19, K703, K709, K73, K746, K754)	3
Peptic ulcer disease	K25, K26, K27, K28	1
Any malignancy including leukemia and lymphoma	C0, C1, C2, C3, C40, C41, C43, C45, C46, C47, C48, C49, C5, C6, C7, C80, C81, C82, C83, C84, C85, C86, C88, C89, C9	2[c]
Metastatic cancer	C77, C78, C79, C80	6
HIV/AIDS	B20, B21, B22, B23, B24, F024, O987, R75, Z219, Z717	6

Note. Main and secondary diagnoses are included. Number in brackets refers to weighting of disease in Charlson Comorbidity Index score. Adapted from Ludvigsson et al. (2). Please refer to Ludvigsson et al. (2) for details on the syntax to generate the Charlson Comorbidity Index variable. [a]Diabetes is scored 0 if Diabetes with end-stage organ disease is present. [b]Mild liver disease is scored 0 if Moderate of severe liver disease is present. [c]Any malignancy including leukemia and lymphoma is scored 0 if Metastatic cancer is present.

REFERENCES

1. Calderón-Larrañaga A, Vetrano DL, Onder G, et al. Assessing and Measuring Chronic Multimorbidity in the Older Population: A Proposal for Its Operationalization. J Gerontol A Biol Sci Med Sci. 2017;72(10):1417–23.
2. Ludvigsson JF, Appelros P, Askling J, et al. Adaptation of the Charlson Comorbidity Index for Register-Based Research in Sweden. Clin Epidemiol. 2021;13:21–41.
